# Tumor-Derived Exosomal Protein Tyrosine Phosphatase Receptor Type O Polarizes Macrophage to Suppress Breast Tumor Cell Invasion and Migration

**DOI:** 10.3389/fcell.2021.703537

**Published:** 2021-09-28

**Authors:** Hongmei Dong, Chaoyu Xie, Yuchen Jiang, Kai Li, Yusheng Lin, Xijiao Pang, Xiao Xiong, Jiehua Zheng, Xiurong Ke, Yexi Chen, Yong Li, Hao Zhang

**Affiliations:** ^1^Institute of Precision Cancer Medicine and Pathology, School of Medicine, Jinan University, Guangzhou, China; ^2^Department of Hematology, University Medical Center Groningen, University of Groningen, Groningen, Netherlands; ^3^Graduate School, Shantou University Medical College, Shantou, China; ^4^Department of Thyroid, Breast and Hernia Surgery, The Second Affiliated Hospital of Shantou University Medical College, Shantou, China; ^5^Laboratory for Translational Surgical Oncology, Department of Surgery, University Medical Center Groningen, University of Groningen, Groningen, Netherlands; ^6^St George and Sutherland Clinical School, Faculty of Medicine, UNSW, Sydney, NSW, Australia; ^7^Cancer Care Centre, St George Hospital, Kogarah, NSW, Australia; ^8^School of Basic Medical Sciences, Zhengzhou University, Zhengzhou, China; ^9^Department of General Surgery, The First Affiliated Hospital of Jinan University, Guangzhou, China

**Keywords:** protein tyrosine phosphatase receptor type O, tumor-derived exosomes, macrophage polarization, breast cancer, invasion and migration

## Abstract

Tumor-derived exosomes, containing multiple nucleic acids and proteins, have been implicated to participate in the interaction between tumor cells and microenvironment. However, the functional involvement of phosphatases in tumor-derived exosomes is not fully understood. We and others previously demonstrated that protein tyrosine phosphatase receptor type O (PTPRO) acts as a tumor suppressor in multiple cancer types. In addition, its role in tumor immune microenvironment remains elusive. Bioinformatical analyses revealed that PTPRO was closely associated with immune infiltration, and positively correlated to M1-like macrophages, but negatively correlated to M2-like macrophages in breast cancer tissues. Co-cultured with PTPRO-overexpressing breast cancer cells increased the proportion of M1-like tumor-associated macrophages (TAMs) while decreased that of M2-like TAMs. Further, we observed that tumor-derived exosomal PTPRO induced M1-like macrophage polarization, and regulated the corresponding functional phenotypes. Moreover, tumor cell-derived exosomal PTPRO inhibited breast cancer cell invasion and migration, and inactivated STAT signaling in macrophages. Our data suggested that exosomal PTPRO inhibited breast cancer invasion and migration by modulating macrophage polarization. Anti-tumoral effect of exosomal PTPRO was mediated by inactivating STAT family in macrophages. These findings highlight a novel mechanism of tumor invasion regulated by tumor-derived exosomal tyrosine phosphatase, which is of translational potential for the therapeutic strategy against breast cancer.

## Introduction

Breast cancer, the most common malignancy among women worldwide, is famous for its high mortality with a large number of patients developing recurrence and metastasis (Oskarsson et al., 2011; Sung et al., 2021). Despite exciting progress in the development of novel therapeutic strategies, such as targeted therapy and immunotherapy, the therapeutic outcome is still unsatisfied and the prognosis remains poor for the patients with metastasis, who have only 26% of estimated 5-year survival (Sambi et al., 2019). Therefore, there is an urgent need to understand and tackle breast cancer metastasis. Metastasis is a multi-step process including the migration and invasion of cancer cells, subsequent proliferation, and colonization in distant organs (Steeg, 2016). It has been well established that the tumor microenvironment (TME) plays an important role in breast cancer cell invasion and metastasis (Cacho-Diaz et al., 2020). However, the molecular mechanisms underlying this process are still poorly understood.

Tumor-associated macrophages (TAMs) in TME are closely associated with tumor immune escape, angiogenesis, tumor proliferation, tumor invasion and migration (Alahari et al., 2015; Zhang S. Y. et al., 2020). The therapeutic strategies targeting TAMs have gradually become valuable for cancer treatment. TAMs typically have high plasticity, either activated pro-inflammatory (M1-like) or alternatively activated immunosuppressive (M2-like) phenotype, as a consequence of diverse stimuli in TME (Komohara et al., 2016). Numerous studies have reported that TAMs mostly present the M2-like phenotype, playing significant roles in promoting invasion and correlating with a poor prognosis in many malignant solid tumors including breast cancer (Rhee, 2016; Choi et al., 2018). However, the mechanisms underlying this reciprocal regulation between cancer cells and TAMs during the invasion process remain unclear.

Exosomes, the small membrane-bound vesicles (30–150 nm in diameter), have been recognized as mediators of intercellular communication between cancer cells and TME by transferring cargos including proteins, DNAs and RNAs between different cell types (Maia et al., 2018; Lin et al., 2019). Increasing evidence suggests that exosomes participate in different processes of cancer formation and progression, including remodeling of TME, angiogenesis, immune escape, dissemination, invasion and metastasis (Liu et al., 2021). Tumor-derived exosomes have been recently implicated in tumor metastatic process via transferring miRNAs or proteins to TAMs (Mantovani et al., 2017; Yuan et al., 2021). Although tumor-derived exosomes are proposed mostly as a pro-tumor factor, they are also referred to an alternative to cell therapy since they are non-living and biocompatible material to transfer genetic cargo to recipient cells. It has been reported that NFAT3-expressing exosomes that are originated from tumor cells inhibit tumor growth and invasion spreading in breast cancer cell-bearing mice (de Camargo et al., 2020). It is likely that tumor-derived exosomes can process either pro-tumor or anti-tumor capacity, depending highly on the nature of the cargos that are delivered by them. The fact that tumor-derived exosomes contain various types of functional tumor-associated proteins, among others, protein tyrosine kinases (PTKs) and protein tyrosine phosphatases (PTPs) has been revealed (Ciravolo et al., 2012; Peinado et al., 2012; Wu et al., 2017; Zhang et al., 2017). However, the functional role of PTPs in tumor-derived exosomes remains unclear.

Protein tyrosine phosphatase receptor type O (PTPRO) belongs to the R3 subtype family of receptor-type protein tyrosine phosphatase. Previously, we reported that the DNA methylation status of PTPRO is a prognostic factor in ERBB2-positive breast cancer (Huang et al., 2013). Besides, we have previously demonstrated that PTPRO inhibited ERBB2-driven breast cancer through dephosphorylation leading to dual effects of ERBB2 signaling suppression and endosomal internalization of ERBB2 (Dong et al., 2017a). PTPRO also plays a critical role in regulating cancer-associated inflammation and anti-tumor immunity (Huang et al., 2018; Jin et al., 2020). A most recent study has shown that increased serum IL-6 downregulated PTPRO expression in human hepatocellular carcinoma (HCC) monocytes and macrophages (Zhang W. et al., 2020). Nevertheless, little is known about the functional involvement of PTPRO expressed in tumor cells in regulating TME.

Given the crucial roles of TAMs and tumor-derived exosomes in dictating cancer invasion, we speculated that the crosstalk between TAMs and tumor cells via exosomes could promote tumor cell invasion and migration by regulating the TAM polarization. Here, we demonstrated that tumor derived PTPRO-expressing exosomes induced macrophages to differentiate to M1 phenotype, and inhibited breast cancer cell invasion and migration. Meanwhile, we revealed the underlying mechanism of macrophage polarization, which involved dephosphorylation of STATs in macrophages modulated by tumor cell-derived exosomal PTPRO. Our findings highlight a novel mechanism of tumor invasion regulated by tumor-derived exosomal tyrosine phosphatase, holding promise for developing new therapeutics against tumor invasion and migration in breast cancer.

## Materials and Methods

### Cell Culture

Human breast cancer cell lines were obtained from the American Type Culture Collection (ATCC, Manassas, VA, United States). ZR-75-1 and MCF-7 cells were cultured in DMEM/F12 (GIBCO/Invitrogen, Carlsbad, CA, United States) supplemented with 10% fetal bovine serum (FBS) (GIBCO/Invitrogen, Carlsbad, CA, United States). All cells were maintained at 37°C in an incubator containing 5% CO_2_. For transwell chamber-based co-cultures, THP-1 cells (1.5 × 10^5^/well) were plated in the lower compartment in 12-well plates with 10 ng/mL phorbol 12-myristate 13-acetate (PMA), and ZR-75-1 (1 × 10^5^) or MCF-7 (1.5 × 10^5^) were seeded in the top compartment of the transwell membrane (0.4 μm pore size, Corning, New York, United States) after the macrophages’ adhesion. The cells were co-cultured in medium with 10% FBS at 37°C and 5% CO_2_ for 72 h.

### Plasmid Construction and Stable Transfection

The WT full-length PTPRO CDS was cloned into pCR3.1 expression vector. The catalytic site mutant form of PTPRO, a C1136S mutation (CS) was generated by site directed mutagenesis, using a QuikChange Site-Directed Mutagenesis kit (Stratagene) according to the manufacturer’s instructions. Lentiviral pGIPZ shRNA vectors targeting human PTPRO (pGIPZ-shPTPRO and V2LHS_226171) and non-targeting pGIPZ control vector (pGIPZ-shCtrl) were obtained from Open Biosystems (Huntsville, AL, United States).

To induce stable cells overexpressing PTPRO and PTPRO-CS, ZR-75-1 and MCF-7 cell were transfected with the plasmid DNA of pCR3.1-PTPRO, pCR3.1-PTPRO-CS or the control plasmid pCR3.1. To generate stable cells silencing PTPRO expression, ZR-75-1 cells were transfected with the plasmid DNA of pGIPZ-shPTPRO or pGIPZ-shCtrl. All transfections were performed with Lipofectamine 3000 (Invitrogen) according to the manufacturer’s instructions.

### Immunofluorescence

Immunofluorescence staining was performed as previously described (Feng et al., 2014; Dong et al., 2017b; Wang et al., 2019, 2020). Briefly, cells were grown on microscope cover glasses and treated as indicated. Subsequently, the cells were fixed with 4% paraformaldehyde, blocked with blocking buffer (3% BSA and 0.2% Triton X-100 in PBS), and incubated with primary antibodies overnight at 4°C. Antibodies used in this study are described in the Supplementary Table 1. The cells were then washed with washing buffer (0.2% BSA and 0.05% Triton X-100 in PBS) and incubated with Alexa Fluor 488-conjugated secondary antibodies for 1 h at room temperature (RT). The cells were stained with DAPI before mounting and imaging on Cytation 5 Cell Imaging Multi-Mode Reader (BioTek, United States).

### Exosomes Extraction and Detection

Exosomes extraction and detection were performed as described previously (Lin et al., 2019). When cells reached a 60% confluence, the culture medium was replaced with FBS-free medium and cultured for 48 h, and then the culture supernatant was collected. Differential ultracentrifugation was used to extract exosomes from the supernatants. In short, centrifugal force of 1,500 × *g* and 3,000 × *g* was used to centrifuge the cell culture supernatant to get rid of cell debris and dead cells. The resulting supernatant was filtered through a 0.2-μm filter. ExoQuick^*TM*^ Kit (System Biosciences, United States) was applied to isolate and enrich exosomes from medium according to the manufacturer’s instructions. Exosomes pellet was resuspended in 100 μl of PBS for further experiments.

To determine the characteristics of exosomes, specific surface markers Alix, TSG101, CD63, CD9, and calnexin were accessed by immunoblotting. The size distribution of exosomes was determined by a NanoSight LM10 system (NanoSight Ltd., Amesbury, United Kingdom). The exosomes were then allowed to settle on copper grids coated with formvar and carbon. Copper grids were immersed in 2% phosphotungstic acid for 1 min. The morphology of exosomes was analyzed under a transmission electron microscope (TEM) (JEM-1400, Hitachi, Shiga, Japan).

### *In vitro* Migration and Invasion Assays

Cell migration and invasion assays were performed using 24-well plates and 8 μm transwell inserts (Corning Life Sciences, Tewksbury, MA, United States). Briefly, for migration assays, tumor cells (2 × 10^4^) suspended in 200 μL serum-free medium were seeded into the upper chamber, and 1 × 10^4^ macrophages in 800 μL medium containing 10% FBS were added to the lower chamber for co-culture. For the invasion assay, the insert membranes were coated with matrigel (50 μL/well) (BD Biosciences, CA, United States) before adding the cells. After a 24 h incubation, cells invading the lower chamber were then stained with 0.1% crystal violet for 30 min and counted from five randomly chosen microscopes fields for each group. Three independent experiments were carried out in triplicate.

### Immunoblotting

Immunoblotting was performed as described previously (Gan et al., 2016; Du et al., 2019; Xiong et al., 2020; Wang et al., 2021b). Briefly, total proteins were extracted from the breast cancer cells or exosomes on ice with radioimmunoprecipitation assay (RIPA) cell lysis buffer supplemented with protease inhibitors. Protein concentrations were quantified by the BCA method. Cell lysates were separated by using 12% sodium dodecyl sulfate-polyacrylamide gel electrophoresis (SDS-PAGE) and then the proteins were transferred onto the polyvinylidene difluoride (PVDF) membrane. The membranes were blocked with 5% skim milk in Tris–buffered saline with Tween-20 (TBST) for 2 h at RT. Subsequently, the membranes were incubated with primary antibodies overnight at 4°C. Antibodies used in this study are described in the Supplementary Table 1. Then, the membranes were washed twice with TBST solution for 10 min each, incubated with horseradish enzyme-labeled secondary antibody for 1 h at RT. An enhanced chemiluminescence (iBright FL1000, Life Technologies, United States) detection system was applied to detect protein signal. The intensity of the protein bands were quantified by densitometry using ImageJ software (v1.8.0q)^1^ (Li et al., 2019).

### qRT-PCR

Real-Time PCR was performed as described previously (Zhang et al., 2013; Wang et al., 2021a). RNA was retro-transcribed using the FastQuant RT Kit (Tiangen, Beijing, China) following total RNA purification with TRIzol (Invitrogen). Specific quantitative real-time PCR experiments were performed using the GoTaq qPCR Master Mix (TAKARA, JAPAN), according to the manufacturer’s instructions. All reactions were processed in triplicate, and GAPDH was selected for normalizing mRNA expression.

Primers used for quantitative real-time PCR were:

IL-1β: sense, 5′-ATGATGGCTTATTACAGTGGCAA-3′,
Antisense, 5′-GTCGGAGATTCGTAGCTGGA-3′;
TNF-α: sense, 5′-CCCCAGGGACCTCTCTCTAATC-3′,
Antisense, 5′-GGTTTGCTACAACATGGGCTACA-3′;
TGF-β: sense, 5′-ACCAACTACTGCTTCAGCTCCA-3′,
Antisense, 5′-GATCATGTTGGACAACTGCTCC-3′;
IL-10: sense, 5′-GACTTTAAGGGTTACCTGGGTTG-3′,
Antisense, 5′-TCACATGCGCCTTGATGTCTG-3′;
GAPDH: sense, 5′-TGCACCACCAACTGCTTAGC-3′,
Antisense, 5′-GGCATGGACTGTGGTCATGAG-3′.

### Statistical Analyses

All statistical analyses were performed using the SPSS 19.0 statistical software package (SPSS Inc., Chicago, IL, United States). The comparisons of data with normal distributions between two groups were performed with Student’s *t*-tests, and those among more than two groups with one-way ANOVA with *post hoc* intergroup comparisons. The relationships of immune scores and PTPRO expression were analyzed by Spearman correlation. A *P-*value < 0.05 was considered statistically significant, except when adjustment for multiple comparisons was needed.

## Results

### Protein Tyrosine Phosphatase Receptor Type O Is Associated With Macrophage Infiltration in Breast Cancer

**FIGURE 1:**
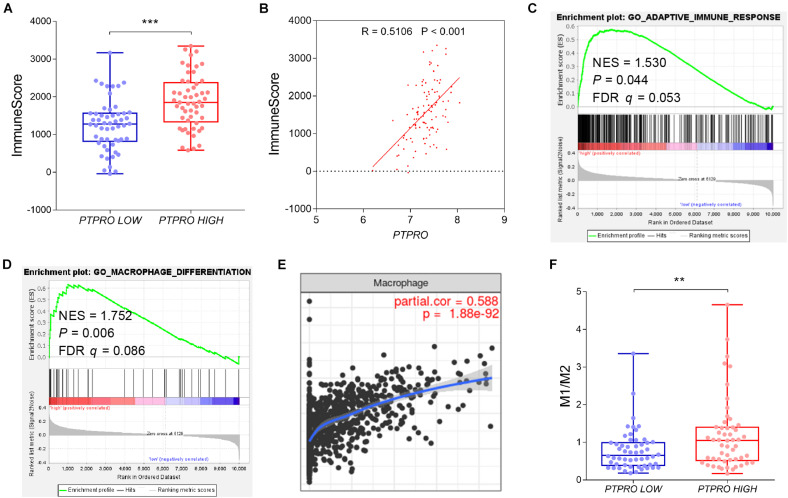
PTPRO expression was associated with TAM infiltration in breast cancer. **(A)** Box and scatter plots showing the immune scores of PTPRO high expression group and PTPRO low expression group in the GEO dataset GSE36774. **(B)** Correlation between PTPRO expression and immune scores in the GEO dataset GSE36774. **(C)** GSEA showing the positive correlation between PTPRO expression and adaptive immune response signature in the GEO dataset GSE36774. **(D)** GSEA showing the positive correlation between PTPRO expression and macrophage differentiation-related gene signature in the GEO dataset GSE36774. **(E)** The correlation between PTPRO expression and the infiltration level of macrophages was presented by Tumor Immune Estimation Resource (TIMER; cistrome.shinyapps.io/timer). **(F)** Box and scatter plots showing M1/M2 ratio difference between PTPRO high expression group and PTPRO low expression group according to GEO dataset GSE36774. FDR *q*, false-discovery rate *q*-value; NES, normalized enrichment score; **P < 0.01, ***P < 0.001 by Student’s *t*-test.

To investigate the impact of PTPRO on TME, we systematically analyzed the correlation of PTPRO and immune infiltration using multiple public breast cancer datasets. PTPRO expression was shown to be positively correlated with immune score estimating by the ESTIMATE algorithm (Figures 1A,B and Supplementary Figures 1A,B). Gene Set Enrichment Analyses (GSEA) revealed that PTPRO expression was positively associated with adaptive immune response gene signature and macrophage differentiation-related gene signature (Figures 1C,D and Supplementary Figures 1C,D). Of note, PTPRO expression was also positively correlated with infiltrating levels of macrophages, which was analyzed using Tumor Immune Estimation Resource (TIMER)^2^ (*r* = 0.588, *P* < 0.001, Figure 1E). To further clarify the relationship between PTPRO level and the abundance of infiltrating immune cells, CIBERSORT algorithm was used to analyze the transcriptomic profiles of the same dataset. We found that patients with high PTPRO expression (higher than median expression level of PTPRO) had higher M1-like, while lower M2-like macrophage infiltrations, resulting in a higher M1-to-M2 ratio (*P* = 0.003, Figure 1F). Taken together, these findings indicate that PTPRO may be involved in polarization of TAMs.

### Tumor Cell-Derived Exosomal Protein Tyrosine Phosphatase Receptor Type O Promotes M1-Like Macrophage Polarization

**FIGURE 2:**
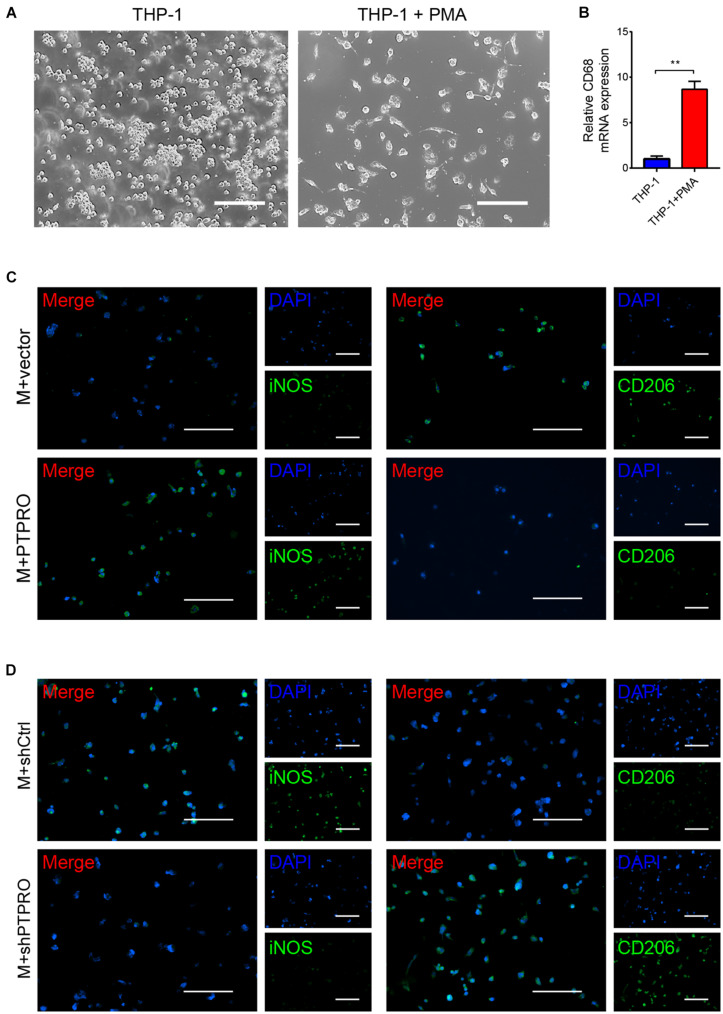
Tumor-derived PTPRO reeducated macrophages toward a M1-like phenotype. **(A)** THP-1 cells were treated with PMA for 24 h. Representative images of THP-1 derived macrophages were shown. Scale bars = 50 μm. **(B)** qRT-PCR was used to detect the expression of CD68 (macrophage marker) in THP-1 cells. **(C)** Representative images of immunofluorescence staining of iNOS and CD206 in THP-1 derived macrophages after incubating with ZR-75-1-vector or ZR-75-1-PTPRO cells for 48 h. Scale bars = 50 μm. **(D)** Representative images of immunofluorescence staining of iNOS and CD206 in THP-1 derived macrophages after incubating with ZR-75-1-shCtrl or ZR-75-1-shPTPRO cells for 48 h. Scale bars = 50 μm. Error bars, SEM. **P < 0.001 by Student’s *t*-test.

To determine the effects of PTPRO expressed in cancer cells on macrophage polarization, we first generated stable PTPRO-overexpressing and PTPRO-knock down breast cancer cells, ZR-75-1-PTPRO and ZR-75-1-shPTPRO, respectively (Supplementary Figures 2A,B). Next, THP-1 monocytes were differentiated into macrophages with PMA. After 24-h treatment of PMA, THP-1 cells became adherent and had upregulated level of macrophage marker CD68, compared to cells that were cultured in the absence of PMA (Figures 2A,B). THP1-derived macrophages were then co-cultured with ZR-75-1 cells using transwell chamber-based model, with macrophages on the lower compartment while cancer cells on the top compartment. Immunofluorescence analyses showed that PTPRO significantly increased the proportion of iNOS^+^ M1-like TAMs while decreased the CD206^+^ M2-like TAMs (Figure 2C), while PTPRO deficiency decreased the proportion of iNOS^+^ TAMs and increased the CD206^+^ TAMs (Figure 2D).

**FIGURE 3:**
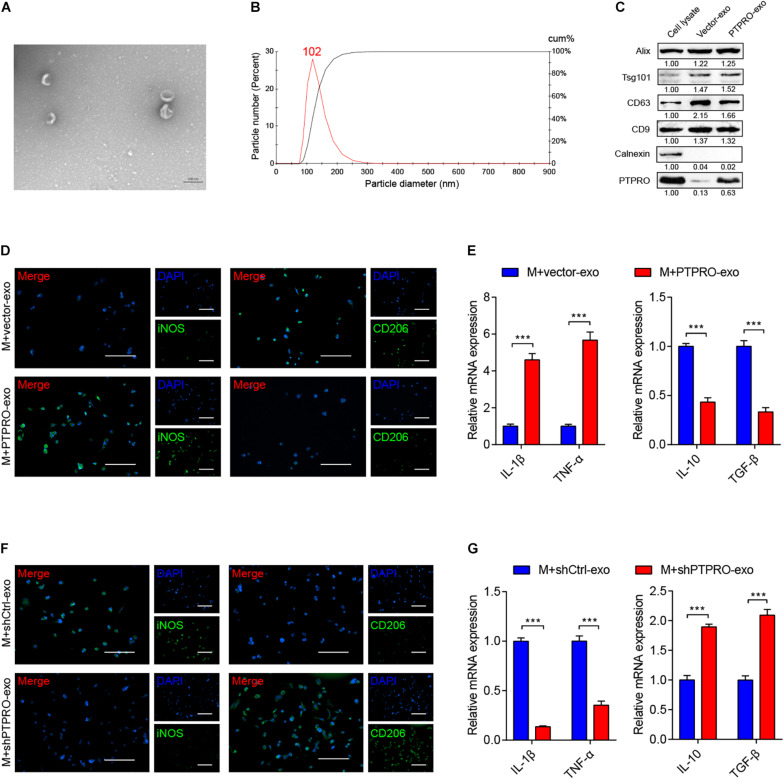
Tumor cell-derived PTPRO inflected macrophage polarization via exosome carrier. **(A)** Electron microscopy images of exosomes isolated from ZR-75-1-PTPRO cells. **(B)** Nanoparticle tracking analysis (NTA) of exosomes isolated from ZR-75-1-PTPRO cells. **(C)** The expressions of Alix, TSG101, CD63, CD9, Calnexin, and PTPRO were measured by immunoblotting in exosomes isolated from ZR-75-1-PTPRO cells. Relative protein expression was quantified. **(D)** Representative images of immunofluorescence staining of iNOS and CD206 in THP1-derived macrophages after treating with ZR-75-1-vector-exo or ZR-75-1-PTPRO-exo for 48 h. Scale bars = 50 μm. **(E)** qRT-PCR was applied to detect the relative expression of IL-1β, TNF-α, IL-10, and TGF-β in THP-1 derived macrophage after treating with ZR-75-1-vector-exo or ZR-75-1-PTPRO-exo for 48 h. **(F)** Representative images of immunofluorescence staining of iNOS and CD206 in THP1-derived macrophages after treating with ZR-75-1-shCtrl-exo or ZR-75-1-shPTPRO-exo for 48 h. Scale bars = 50 μm. **(G)** qRT-PCR was applied to detect the relative expression of IL-1β, TNF-α, IL-10, and TGF-β in THP-1 derived macrophage after treating with ZR-75-1-shCtrl-exo or ZR-75-1-shPTPRO-exo for 48 h. Error bars, SEM. ***P < 0.001 by Student’s *t*-test.

In the previously described co-culture experiment, being plated in different compartments eliminated gap junction-mediated intercellular communication between cancer cells and macrophages. However, the transwell membrane allows the diffusion of small vesicles including exosomes between separated populations of cells. As increasing studies have shown that tumor cell-derived exosomes may epigenetically reprogram surrounding cells via delivery of functional proteins, DNAs, and RNAs (Baig et al., 2020). Therefore, we hypothesized that PTPRO regulates macrophage polarization via exosome-mediated intercellular communication. Exosomes were isolated from ZR-75-1-PTPRO and ZR-75-1-vector cells, as well as ZR-75-1-shPTPRO and ZR-75-1-shCtrl cells. As visualized by TEM, the isolated exosomes have the typical size of exosomes (30–150 nm) and the morphology of bilayer cup shape as exhibited representatively (Figure 3A). Nanoparticle tracking analysis (NTA) indicated that most of the particles had a diameter between 80 nm and 150 nm with a peak at around 102 nm (Figure 3B). Additionally, immunoblotting revealed that the vesicles were positive for exosomes markers Alix, Tsg101, CD63, and CD9 but negative for endoplasmic reticulum marker calnexin (Figure 3C). Furthermore, immunoblotting demonstrated that PTPRO was enriched in exosomes derived from PTPRO overexpressing cells, indicating that PTPRO could be encapsulated in exosomes delivered from breast cancer cells (Figure 3C).

To explore the interaction between tumor-derived exosomes and macrophages *in vitro*, we incubated THP1-derived macrophages with ZR-75-1-derived exosomes. As shown in Figure 3D, the number of iNOS^+^ M1-like TAMs was significantly increased, and the percentage of CD206^+^ M2-like TAMs was relatively decreased in macrophages incubated with ZR-75-1-PTPRO cell-derived exosomes (PTPRO-exo) compared to ZR-75-1-vector cell-derived exosomes (Vector-exo), while in PTPRO knockdown cells, the percentage of iNOS^+^ M1-like TAMs was reduced and CD206^+^ M2-like TAMs was significantly increased (Figure 3F). Furthermore, qRT-PCR assay revealed that PTPRO-exo addition significantly increased the expression of IL-1β and TNF-α, but reduced the expression of TGF-β, IL-10 in macrophages (Figure 3E). Incubation with exosomes derived from PTPRO knockout cells (shPTPRO-exo) significantly decreased the expression of IL-1β and TNF-α, but increased the expression of TGF-β, IL-10 in macrophages (Figure 3G). Taken together, the data demonstrated that breast cancer cell-derived exosomal PTPRO induced macrophages to differentiate toward M1-like phenotype.

### Tumor Cell-Derived Exosomal Protein Tyrosine Phosphatase Receptor Type O Inhibits Cancer Cell Invasion and Migration

**FIGURE 4:**
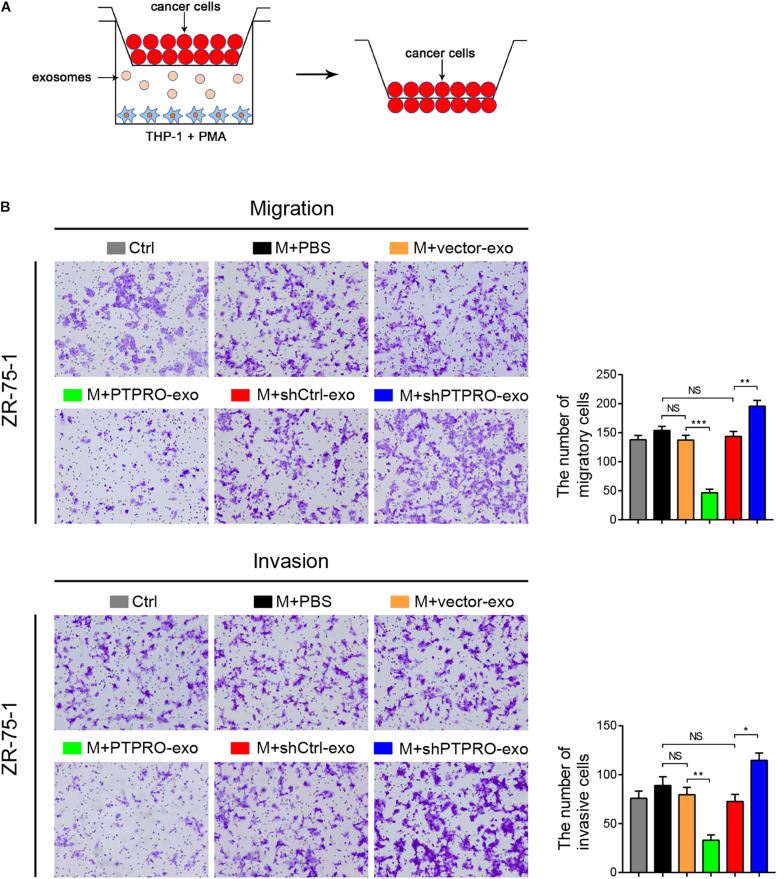
Tumor cell-derived exosomal PTPRO inhibited cancer cell invasion and migration. **(A)** Schematic illustration of the *in vitro* transwell co-culture system. **(B)** Cell migration and invasion assays were used to study the migration and invasion ability of ZR-75-1 cells. THP-1 cells prestimulated with PMA were treated with ZR-75-1-vector-exo or ZR-75-1-PTPRO-exo and ZR-75-1-shCtrl-exo or ZR-75-1-shPTPRO-exo at a concentration of 25 μg/mL or PBS; then, the upper chamber was obtained and incubated with ZR-75-1 cells for 24 h. Original magnification: 200×. Error bars, SEM. *P < 0.05; **P < 0.01; ***P < 0.001 by a one-way ANOVA with *post hoc* intergroup comparisons.

We next used transwell assays to determine whether the switching of macrophage phenotype driven by PTPRO-exo is responsible for preventing migration and invasion of breast cancer cells (Figure 4A). The transwell migration and invasion assays revealed that compared with the control groups, THP1-derived macrophages (pre-stimulated with PMA) and stimulated by PTPRO-exo could significantly inhibit the migration and invasion abilities of ZR-75-1 cells (Figure 4B). Consistent results were observed in another breast cancer cell line MCF-7 (Supplementary Figures 3A,B). Moreover, the migration and invasion abilities of ZR-75-1, which were treated with exosomes derived from ZR-75-1-shPTPRO cells, were significantly increased compared with those in the control groups (Figure 4B). These results indicate that tumor cell-derived exosomal PTPRO inhibited the invasion and migration abilities of breast cancer cells.

### Tumor Cell-Derived Exosomal Protein Tyrosine Phosphatase Receptor Type O Induces M1 Polarization of Macrophages via STAT3/STAT6 Signaling

**FIGURE 5:**
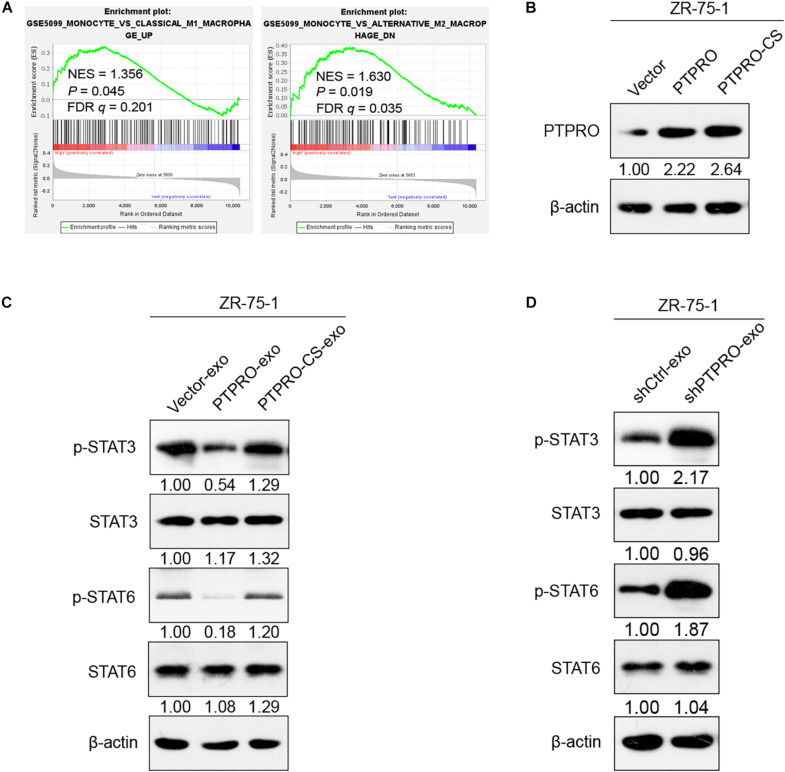
Tumor cell-derived exosomal PTPRO polarized the M1 macrophage via dephosphorylated STAT3/STAT6 signaling. **(A)** GSEA showing inverse correlation between STAT3 expression and M1-gene signature (MONOCYTE_VS_CLASSICAL_M1_MACROPHAGE_UP) and positive correlation between STAT6 expression and M2-gene signature (MONOCYTE_VS_ALTERNATIVE_M2_MACROPHAGE_DN) in a published cohort of breast cancer. **(B)** Immunoblotting revealed that PTPRO was efficiently over-expressed in ZR-75-1 cells and the mutation of PTPRO did not affect it’s protein expression level. Relative protein expression was normalized to β-actin. **(C)** The expressions of STAT3/STAT6 protein and corresponding phosphorylated protein in THP-1 derived macrophage after treating with ZR-75-1-vector-exo, ZR-75-1-PTPRO-exo or ZR-75-1-PTPRO-CS-exo. Relative protein expression was normalized to β-actin. **(D)** The expressions of STAT3/STAT6 protein and corresponding phosphorylated protein in THP-1 derived macrophage after treating with ZR-75-1-shCtrl-exo or ZR-75-1-shPTPRO-exo. Relative protein expression was normalized to β-actin. FDR *q*, false-discovery rate *q*-value; NES, normalized enrichment score.

STAT3 and STAT6 are the crucial transcriptional factors involved in promoting macrophages polarization into a M2-like phenotype (Li et al., 2018). GSEA indicated that STAT3 expression was inversely associated with M1-like macrophages-related gene signature (*P* = 0.045, Figure 5A, left panel), and STAT6 expression was positively associated with M2-like macrophages-related gene signature (*P* = 0.019, Figure 5A, right panel). To investigate whether STAT3 and STAT6 are involved in macrophage polarization regulated by PTPRO-exo, and further investigate whether the phosphorylation of STAT3 and STAT6 were regulated by the dephosphorylate function of PTPRO, we mutated the catalytic site (CS) of PTPRO and transfected this plasmid into ZR-75-1 and MCF-7 cell lines (Figure 5B and Supplementary Figure 4A). Immunoblotting indicated that PTPRO-exo, but not vector-exo, reduced phospho-STAT3 and phospho-STAT6 protein levels in THP-1-derived macrophages. Moreover, exosomes derived from PTPRO mutation cells (PTPRO-CS-exo) did not affect the phospho-STAT3 and phospho-STAT6 protein levels significantly in THP-1-derived macrophages (Figure 5C and Supplementary Figure 4B). Therefore, PTPRO phosphatase activity is required for its impact on STAT3/STAT6 signaling in macrophages. In addition, exosomes derived from PTPRO knockout cells enhanced STAT3/STAT6 signaling in THP-1-derived macrophages (Figure 5D). Taken together, these results demonstrated that tumor cell-derived exosomal PTPRO promoted macrophages to differentiate into M1-like phenotype likely by inactivating the STAT signaling with its dephosphorylate function.

## Discussion

In this study, we showed that PTPRO plays a role in immune infiltration in breast cancer. Further, we confirmed that tumor-derived exosomal PTPRO induced macrophages to switch into a M1-like phenotype, leading to decreased migration and invasion abilities of tumor cells. Dephosphorylation of STAT3 and STAT6 was involved in exosomal PTPRO mediated M1-like macrophage polarization. Our results unveil a novel function of tumor-derived exosomal tyrosine phosphatase in breast cancer invasion and migration by modulating macrophage polarization.

Protein tyrosine phosphatase receptor type O has been recognized as a tumor suppressor in multiple cancer types (Motiwala et al., 2004; You et al., 2012; Dong et al., 2017a). Previous studies have paid more attentions to the influence of endogenous factors of tumor cells on mammary tumor progression and metastasis, it is now well-recognized that the TME provides essential support for malignant phenotype of tumor cells and also evolves along with tumor development (Gibby et al., 2012). A recent study revealed that PTPRO deficiency results in a reduction of NF-κB activation in both hepatocytes and macrophages in the TME, which is related to c-Src phosphorylation (Jin et al., 2020). Loss of PTPRO in the tumor niche is correlated with tumor metastases of breast cancer (Liu et al., 2015). In addition, the deletion of PTPRO promotes PD-L1 secretion in both monocytes and macrophages through JAK2/STAT1 and JAK2/STAT3/c-MYC pathways (Zhang W. et al., 2020). Together with the finding that PTPRO expression is correlated with the activation of immune response in human clear cell renal cell carcinoma (Gan and Zhang, 2020), the potential role of PTPRO in immune regulation has been noticed. In line with these studies, we found that PTPRO is closely correlated with TAMs. TAMs with the high flexibility and plasticity could be polarized to M1-like or M2-like subtypes depending on diverse environmental factors in the TME (He et al., 2020). In triple-negative breast cancer (TNBC), cancer cell-derived IL-6 have been reported to stimulate the polarization of macrophages toward M2 phenotype, and thus promotes epithelial-mesenchymal transition (EMT) and invasion of TNBC cells (Weng et al., 2019). By evaluating the association between PTPRO and macrophage infiltration, we found that PTPRO increased M1-like TAMs, while decreased M2-like TAMs in breast cancer.

Cell invasion and migration are finely regulated processes that are critical in cancer progression and metastasis (Entschladen et al., 2004). Not only the driving signaling in tumor cells, the crosstalk between tumor cells and TME is also responsible for tumor cell invasion and migration (Neophytou et al., 2021). Tumor cells, TAMs, and other stromal cells within TME communicate with each other through the secretion of exosomes, carrying various molecules. It has been reported that exosomes derived from breast cancer cells could promote M2-like polarization and enhance its tumor-promoting function by transmitting lncRNA BCRT1 (Liang et al., 2020). TNBC cell-derived exosomes have been indicated as a factor in the induction of M2-like macrophage polarization which benefits breast cancer cells *in vitro*, and supports tumor growth and axillary lymph node metastasis in the orthotopic TNBC mouse model (Piao et al., 2018).

Several lines of evidence proved the presence of protein tyrosine phosphatases (PTPs) in exosomes (Coren et al., 2008; Moratti et al., 2015; Wu et al., 2017). For example, it has been found that melanoma exosomes containing both protein and mRNA of PTPN11 could be efficiently delivered and dose-dependently increase PTPN11 abundance in T cells (Wu et al., 2017). However, the impact of tumor-derived exosomal PTPs on TAMs remains unclear. In the present study, we found that tumor-derived exosomal PTPRO triggered M1-like macrophage polarization to suppress the migration and invasion of breast cancer cells. The transcriptional regulation of macrophage polarization has become the focus of numerous studies in recent years. For example, STAT1 and NF-κB are important transcription factors involved in the polarization of M1-like macrophage, whereas STAT3, STAT6, KLF4, GATA3, and c-MYC are associated with the process of M2-like macrophage polarization (Li et al., 2018). A previous finding has shown that exosomes secreted by breast cancer cells can skew macrophage polarization toward a pro-tumoral M2-like phenotype partially via gp130/STAT3 signaling (Ham et al., 2018), suggesting that the tumor-derived exosomes mediate immune-suppressive activity of macrophages. In contrast, we demonstrated that tumor-derived exosomal PTPRO promotes macrophages to differentiate into M1-like macrophages, resulting in inhibition of tumor cell invasion and migration. Mechanistically, exosomal PTPRO induced STAT3 and STAT6 inactivation in macrophages, which is responsible for their phenotype switching from M2 to M1. It was recently reported that PTPRO expression in macrophages can be decreased by increased level of IL-6 in serum from patients with HCC (Zhang W. et al., 2020). However, whether the alteration of PTPRO in macrophage can lead to phenotype switching from M2 to M1 is unknown. To elucidate this question, a mouse model with knock-out of macrophage-specific expression of PTPRO is required, which is clearly beyond the scope of the current report. Our study highlights the critical role of exosomal PTPRO in suppressing breast cancer invasion and migration.

We focus on TAMs throughout the current study based on the correlation of PTPRO expression and macrophage infiltration that we found in patients with breast cancer. Besides TAMs, TME contains a host of other cell types, including T cells, B cells, dendritic cells (DC), natural killer (NK) cells, granulocytes, mast cells, vascular endothelial cells, adipocytes as well as fibroblasts. Whether PTPRO has impacts on these cell types warrant further study. Additionally, the exact cargos from tumor-derived exosomes were not well identified except for PTPRO in the current study, future efforts for assessing the exosomal transcripts and proteins which are responsible for malignant phenotype are warranted. Importantly, more researches using animal models and patient samples are warranted to validate our findings.

In summary, our studies showed that tumor cell-derived exosomal PTPRO inhibits invasion and migration abilities of breast cancer cells via modulating TAM polarization. These findings broaden our understanding of the mechanisms of tumor cell invasion and migration that is regulated by tumor-derived exosomal tyrosine phosphatase PTPRO, which could be used as a potential therapeutic target for breast cancer.

## Data Availability Statement

The raw data supporting the conclusions of this article will be made available by the authors, without undue reservation.

## Author Contributions

HZ and HD: study design and funding support. CX, YJ, KL, XX, and XP: cellular experiments. HD, CX, YJ, YSL, JZ, and YC: data collection and analyses. HD, YJ, YL, and XK: writing and revision of the manuscript. HZ: supervision. All authors contributed to the work and approved it for publication.

## Conflict of Interest

The authors declare that the research was conducted in the absence of any commercial or financial relationships that could be construed as a potential conflict of interest.

## Publisher’s Note

All claims expressed in this article are solely those of the authors and do not necessarily represent those of their affiliated organizations, or those of the publisher, the editors and the reviewers. Any product that may be evaluated in this article, or claim that may be made by its manufacturer, is not guaranteed or endorsed by the publisher.

## Footnotes

1
http://rsb.info.nih.gov/ij

2
cistrome.shinyapps.io/timer
