# Supplementary material for: Tumor-Derived Exosomal Protein Tyrosine Phosphatase Receptor Type O Polarizes Macrophage to Suppress Breast Tumor Cell Invasion and Migration
Source: Front Cell Dev Biol. 2021 Sep 28;9:703537. doi: 10.3389/fcell.2021.703537 (PMC8505750; doi:10.3389/fcell.2021.703537)
Supplement: Supplementary file 1 [file Data_Sheet_1.pdf]

Supplementary information

Supplementary Materials and Methods

Immune Score Analysis for the Microenvironment

To assess the possible association of immune score with PTPRO expression in human primary breast cancer, two expression cohorts from primary breast tumors were obtained from the Gene Expression Omnibus (GEO) database (n = 256 for GSE36774, n = 97 for GSE81954). The immune score was calculated by using the ESTIMATE algorithm to the expression matrix (1). Furthermore, the cases were assigned to high and low PTPRO expression groups based on the median value of PTPRO expression values, to identify the possible association of immune score with PTPRO expression.

Gene Set Enrichment Analyses

To further explore the potential relationship between PTPRO expression with organism immune, the mRNA profiles of primary breast cancer from GSE36774 (n = 256) and GSE81954 (n = 97) were processed and subjected to the Gene Set Enrichment Analyses (GSEA) (2), using GSEA software (version 4.1.0). Next, to verify the relationship between STAT3, STAT6 expression with macrophage polarization, the mRNA profiles of primary breast cancer from GSE82173 were processed to GSEA.

TIMER Database Analysis

The correlation of PTPRO expression with the abundance of macrophages infiltrating in breast cancer was analyzed in Tumor Immune Estimation Resource (TIMER; cistrome.shinyapps.io/timer) (3).

Analysis of the Relative Proportions of Macrophages (M0, M1, M2) in Breast Cancer

Macrophages in breast cancer samples from the GEO cohort (GSE36774, GSE81954) were assessed by using the CIBERSORT deconvolution algorithm. The gene expression matrix of macrophages was performed from the CIBERSORT platform (https://cibersortx.stanford.edu/). The results of the inferred proportions of macrophages assessed by CIBERSORT were considered to be accurated at a threshold of $P<0.05$ (4). Consequently, only samples with a CIBERSORT $P<0.05$ were qualified for further analysis. Furthermore, the number of permutations of the default signature matrix was set to 100.

Reference

1. Yoshihara K, Shahmoradgoli M, Martinez E, Vegesna R, Kim H, Torres-Garcia W *et al*: Inferring tumour purity and stromal and immune cell admixture from expression data. *Nat Commun* (2013) 4:2612. doi: 10.1038/ncomms3612
2. Subramanian A, Tamayo P, Mootha VK, Mukherjee S, Ebert BL, Gillette MA *et al*: Gene set enrichment analysis: a knowledge-based approach for interpreting genome-wide expression profiles. *Proc Natl Acad Sci U S A* (2005) 102(43):15545-15550. doi: 10.1073/pnas.0506580102
3. Li T, Fan J, Wang B, Traugh N, Chen Q, Liu JS, Li B, Liu XS: TIMER: A Web Server for Comprehensive Analysis of Tumor-Infiltrating Immune Cells. *Cancer Res* (2017) 77(21):e108-e110. doi: 10.1158/0008-5472.CAN-17-0307
4. Ali HR, Chlon L, Pharoah PD, Markowetz F, Caldas C: Patterns of Immune Infiltration in Breast Cancer and Their Clinical Implications: A Gene-Expression-Based Retrospective Study. *PLoS Med* (2016) 13(12):e1002194. doi: 10.1371/journal.pmed.1002194

Supplementary Table

Supplementary Table S1. Antibodies used in this study

Antibody	Application	Dilution	Host	Source	Cat. #
iNOS	IF	1:100	Rabbit Ab	Abcam, Cambridge, UK	ab15323
CD206	IF	1:2000	Rabbit Ab	Abcam, Cambridge, UK	ab64693
Alix	WB	1:1000	Rabbit Ab	Cell Signaling Technology, MA, USA	ab117600
TSG101	WB	1:1000	Rabbit Ab	Abcam, Cambridge, UK	ab133586
CD63	WB	1:1000	Rabbit Ab	Abcam, Cambridge, UK	ab134045
CD9	WB	1:1000	Rabbit Ab	Abcam, Cambridge, UK	ab223052
Calnexin	WB	1:1000	Rabbit Ab	Cell Signaling Technology, MA, USA	#2433
PTPRO	WB	1:500	Rabbit Ab	Proteintech Group Inc, USA	12161-1-AP
p-STAT3	WB	1:1000	Rabbit Ab	Cell Signaling Technology, MA, USA	#9145
STAT3	WB	1:1000	Mouse Ab	Cell Signaling Technology, MA, USA	#9139
p-STAT6	WB	1:1000	Rabbit Ab	Cell Signaling Technology, MA, USA	#9361
STAT6	WB	1:1000	Rabbit Ab	Cell Signaling Technology, MA, USA	#9362
β-actin	WB	1:2000	Rabbit Ab	Proteintech, Rosemont, USA	#4967

Supplementary Figures and Figure legends

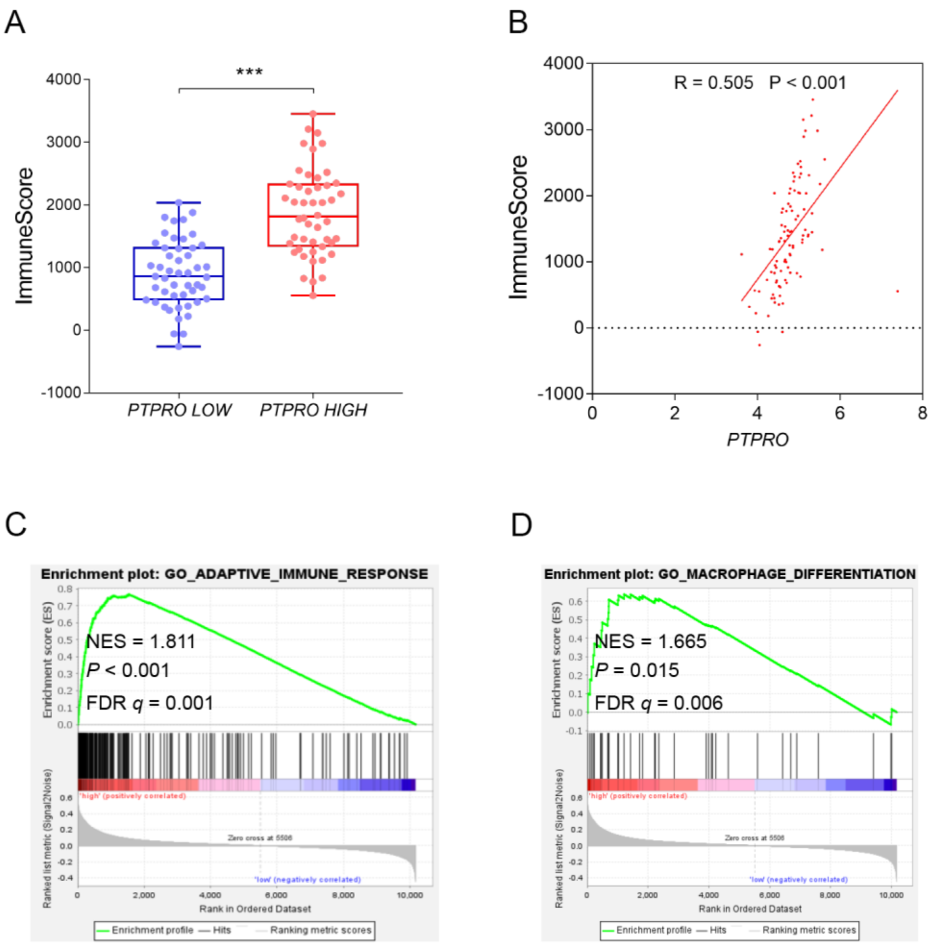

Figure S1. PTPRO expression was associated with immune infiltration in breast cancer. (A) Box and scatter plots showing the immune scores of PTPRO high expression group and PTPRO low expression group in the GEO dataset GSE81954. **(B)** Correlation between PTPRO expression and immune scores in the GEO dataset GSE81954. **(C)** GSEA showing the positive correlation between PTPRO expression and adaptive immune response signature in the GEO dataset GSE82173. **(D)** GSEA showing the positive correlation between PTPRO expression and macrophage differentiation-related gene signature in the GEO dataset GSE82173. FDR q,

false-discovery rate q value; NES, normalized enrichment score; ***P<0.001 by Student's t-test.

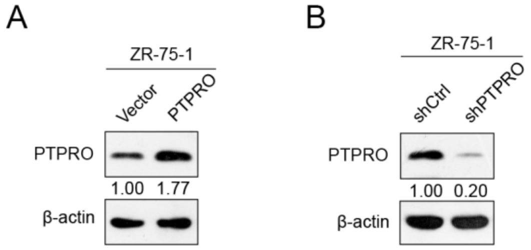

Figure S2. PTPRO expression in ZR-75-1 cells. (A) Immunoblotting revealed that PTPRO was efficiently over-expressed in ZR-75-1 cells. Relative PTPRO protein expression was normalized to β-actin. **(B)** Immunoblotting revealed that PTPRO was efficiently knockdown in ZR-75-1 cells. Relative PTPRO protein expression was normalized to β-actin.

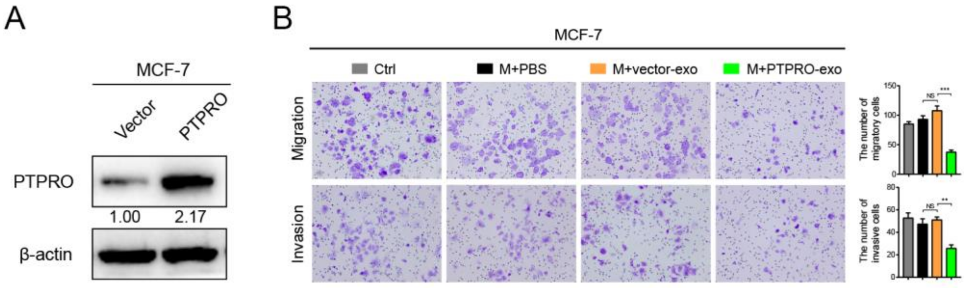

Figure S3. Tumor cell-derived exosomal PTPRO inhibited MCF-7 cells invasion and migration. **(A)** Immunoblotting revealed that PTPRO was efficiently over-expressed in MCF-7 cells. Relative PTPRO protein expression was normalized to β-actin. **(B)** Cell migration and invasion assays were used to study the migration and invasion ability of MCF-7 cells. Original magnification: 200×. Error bars, SEM. $**P < 0.01$; $***P < 0.001$ by a one-way ANOVA with post hoc intergroup comparisons.

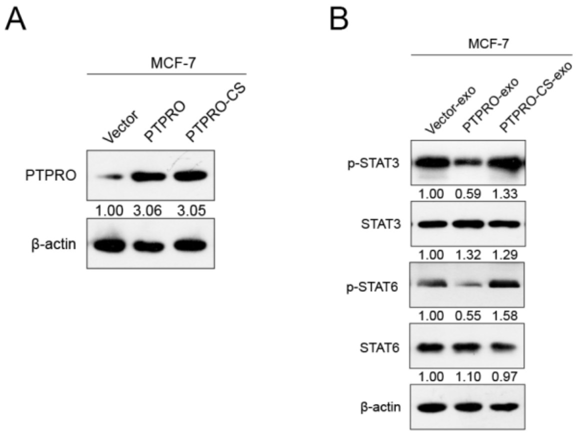

Figure S4. Tumor cell-derived exosomal PTPRO dephosphorylated STAT3/STAT6 signaling. **(A)** Immunoblotting revealed that PTPRO was efficiently over-expressed in MCF-7 cells and the mutation of PTPRO did not affect it's protein expression level. Relative PTPRO protein expression was normalized to β-actin. **(B)** The expressions of STAT3/STAT6 protein and corresponding phosphorylated protein were measured by immunoblotting in THP-1 derived macrophages after treating with MCF-7-vector-exo, MCF-7-PTPRO-exo or MCF-7-PTPRO-CS-exo.
